# Effects of Arachidonic Acid and Its Metabolites on Functional Beta-Cell Mass

**DOI:** 10.3390/metabo12040342

**Published:** 2022-04-12

**Authors:** Karin J. Bosma, Cecilia E. Kaiser, Michelle E. Kimple, Maureen Gannon

**Affiliations:** 1 Department of Veterans Affairs, Tennessee Valley Authority, Nashville, TN 37212, USA; karin.bosma@vumc.org; 2 Department of Medicine, Vanderbilt University Medical Center, Nashville, TN 37232, USA; 3 Department of Medicine, University of Wisconsin-Madison, Madison, WI 53705, USA; cekaiser@wisc.edu; 4 William S. Middleton Memorial Veterans Hospital, Madison, WI 53705, USA; 5 Department of Cell and Regenerative Biology, University of Wisconsin-Madison, Madison, WI 53705, USA; 6 Department of Medicine, Division of Diabetes, Endocrinology, and Metabolism, University of Wisconsin-Madison, 4148 UW Medical Foundation Centennial Bldg., Madison, WI 53705, USA; 7 Department of Molecular Physiology and Biophysics, Vanderbilt University, Nashville, TN 37232, USA; 8 Department of Cell and Developmental Biology, Vanderbilt University, Nashville, TN 37232, USA; 9 Department of Medicine, Division of Diabetes, Endocrinology, and Metabolism, Vanderbilt University Medical Center 2213 Garland Ave., 7465 MRB IV, Nashville, TN 37232-0475, USA

**Keywords:** arachidonic acid, beta-cell mass, eicosanoids, prostaglandins

## Abstract

Arachidonic acid (AA) is a polyunsaturated 20-carbon fatty acid present in phospholipids in the plasma membrane. The three primary pathways by which AA is metabolized are mediated by cyclooxygenase (COX) enzymes, lipoxygenase (LOX) enzymes, and cytochrome P450 (CYP) enzymes. These three pathways produce eicosanoids, lipid signaling molecules that play roles in biological processes such as inflammation, pain, and immune function. Eicosanoids have been demonstrated to play a role in inflammatory, renal, and cardiovascular diseases as well type 1 and type 2 diabetes. Alterations in AA release or AA concentrations have been shown to affect insulin secretion from the pancreatic beta cell, leading to interest in the role of AA and its metabolites in the regulation of beta-cell function and maintenance of beta-cell mass. In this review, we discuss the metabolism of AA by COX, LOX, and CYP, the roles of these enzymes and their metabolites in beta-cell mass and function, and the possibility of targeting these pathways as novel therapies for treating diabetes.

## 1. Introduction

In the United States, diabetes is a major health concern, affecting over 11% of the population and costing an estimated $327 billion annually [1]. Diabetes is characterized primarily by hyperglycemia and is commonly divided into two major types. Type 1 diabetes (T1D) is characterized by autoimmune destruction of the insulin-producing beta cells in the pancreas, resulting in a loss of circulating insulin and subsequent hyperglycemia. Type 2 diabetes (T2D) is primarily associated with obesity and insulin resistance, accompanied by a failure of beta cells to secrete sufficient insulin to overcome the resistance, thus leading to hyperglycemia. Both T1D and T2D can be thought of as diseases of insufficient functional beta-cell mass, and there is great interest in developing therapies to increase beta-cell mass and/or improve beta-cell function.

One potential signaling pathway to target to modulate beta-cell mass or function involves the metabolism of arachidonic acid (AA), a polyunsaturated 20 carbon fatty acid. AA is released from membrane phospholipids through the action of phospholipase A_2_ (PLA_2_), which recognizes and hydrolyzes the *sn*-2 acyl bonds of the phospholipids. PLA_2_ inhibition has been shown to affect beta-cell function. For example, the treatment of rat islets with PLA_2_ inhibitors suppresses glucose-stimulated insulin secretion (GSIS) without affecting glucose metabolism [2] or the activity of other phospholipases [3]. A similar effect was also observed in human islets [4]. This suppression of GSIS could be due to the effect of PLA_2_ inhibition on calcium transients; treatment of mouse islets with a PLA_2_ inhibitor abolished calcium oscillations in response to glucose [5]. In beta cells, PLA_2_ is activated by increases in glucose concentration [6] and adenosine triphosphate (ATP) [7].

While PLA_2_ inhibition has clear effects on GSIS, it is less clear if those effects are due to loss of AA production or to effects on other PLA_2_ substrates. However, in support of a direct role for AA in stimulating GSIS, treatment of isolated human islets with exogenous AA resulted in increased GSIS [4]. Additionally, in clonal rat beta cells, treatment with exogenous AA increased GSIS and also stimulated cell growth [8]. AA has also been shown to protect beta cells against the negative effects of palmitic acid exposure; concomitant incubation of rat beta cells with both AA and palmitic acid led to decreased cell death, increased GSIS, and decreased production of reactive oxygen species (ROS) when compared to cells incubated with palmitic acid alone [9]. This suggests that AA could be beneficial not only for beta-cell function, but also for protecting or increasing beta-cell mass. However, it is not entirely clear in these studies whether the effects seen with AA treatment are due to AA itself, or to its biologically active eicosanoid metabolites (discussed below). The beta-cell response to AA and its metabolites may also be context dependent; for example, islets isolated from both T1D and T2D mouse models fed a diet enriched in eicosapentaenoic acid (EPA), which competes with AA for the same downstream metabolic enzymes, showed an improved GSIS response [10], suggesting that decreased AA metabolism may be protective in the setting of diabetes.

**Figure 1:**
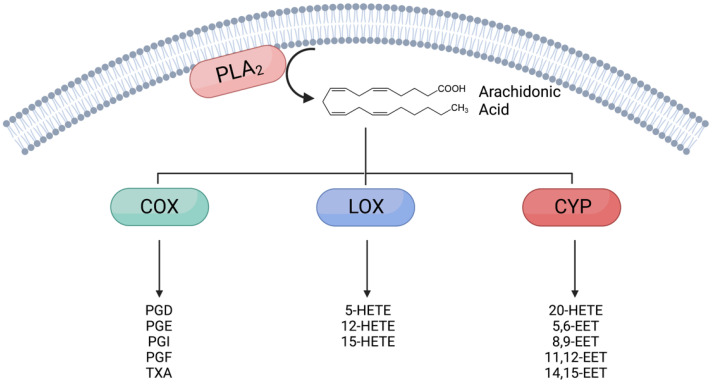
Eicosanoids derived from arachidonic acid (AA). Phospholipids containing AA are hydrolyzed by phospholipase A_2_ (PLA_2_), releasing free AA. AA can be subsequently metabolized by three enzymes and their associated pathways, cyclooxygenase (COX), lipoxygenase (LOX and cytochrome P450 (CYP)). These enzymes mediate the production of the eicosanoids, biologically active metabolites of AA including prostaglandins (PGs), thromboxane (TXA), hydroxyeicosatetraenoic acids (HETEs), and epoxyeicosatrienoic acids (EETs).

AA is primarily metabolized through three major pathways, mediated by the activity of cyclooxygenase (COX), lipoxygenase (LOX), and cytochrome P450 (CYP) enzymes (Figure 1). All three pathways generate eicosanoids (20 carbon fatty acids), which play a role in many biological processes including inflammation [11], immune function [12,13], vascular function [14], and pain perception [15]. This review will focus on the enzymes that metabolize AA in each of these pathways as well as the metabolites generated and their effects on beta-cell mass and function. The effects of treatment with these metabolites on beta cells are summarized in Table 1, and the effects of loss of AA metabolizing enzymes or signaling pathways are summarized in Table 2.

## 2. Lipoxygenase-Derived Molecules (HETEs)

The lipoxygenase (LOX) family of enzymes catalyze the oxygenation of AA and are classified mainly according to which specific carbon atom is oxygenated (typically the 5, 12, and/or 15 positions) (Figure 1). The best characterized lipoxygenases in humans are 5-LOX (encoded by *ALOX5*, also called leukocyte 5-LOX) and 12-LOX (encoded by *ALOX12* or *ALOX15*, also known as platelet 12-LOX and leukocyte 12-LOX, respectively) [38]. Both 5-LOX and 12-LOX generate hydroxyeicosatetraenoic acids (HETEs) from AA. 5-LOX produces 5-HETE from AA as well as many leukotrienes. Platelet 12-LOX converts AA to 12-hydroperoxyeicosatetraenoic acid (12-HPETE), which can be reduced by glutathione peroxidase to the more stable 12-HETE. Similarly, leukocyte 12-LOX (also known as 12/15-LOX) produces 12-HETE, but also produces lesser amounts of 15-HETE, at a ratio of about 6:1 [38]. Lipoxygenase-derived metabolites of AA, regardless of which lipoxygenase produces them, are generally considered to be proinflammatory. Products of 5-LOX have been described to be detrimental in both cardiovascular and renal diseases [39,40,41,42,43].

*Alox5* mRNA has been detected in human islets [4], and 5-LOX products have been detected in rodent islets, although these are present at lower levels than products of 12-LOX [44]. Global *Alox5* deficiency in mice results in impaired insulin secretion despite a trend toward increased beta-cell mass (a result of an increase in individual beta-cell size and number) [26]. Insulin content was also lower in islets from *Alox5* null mice relative to their controls, and consistent with this observation, expression of the *insulin2* gene (*Ins2*) and the critical beta-cell identity transcription factor *Pdx1* was also decreased [26]. siRNA knockdown of *ALOX5* expression in isolated human islets also resulted in decreased *Pdx1* and *insulin* and decreased insulin secretion [26]. These results suggest that 5-LOX is important for the regulation of beta-cell function. However, it is possible that the loss of 5-LOX activity results in the shunting of AA to other pathways, and it is the effects of increased activity of those alternate pathways that lead to the negative effects of 5-LOX deficiency on beta-cell function rather than direct effects of 5-LOX and its AA metabolites. Likewise, it is not clear whether the effects of global 5-LOX deficiency are due to its loss in beta cells or to its loss in other cell types such as macrophages. Further study is also needed to determine which of the AA metabolites produced by 5-LOX might be responsible for effects on beta-cell mass and function.

A role for 12-LOX and its lipid products in pancreas development has been identified in zebrafish. Knockdown of *alox12* expression using antisense morpholinos caused a reduction in overall pancreas size, and a significant decrease in beta-cell number, while alpha-cell number remained unaffected [45]. Knockdown of *gpr31*, the putative receptor for the primary 12-LOX product 12-HETE [46], resulted in a similar reduction in pancreas size and beta-cell number, suggesting that a signaling axis involving 12-LOX, 12-HETE, and GPR31 is required for normal pancreatic development in zebrafish. A role for 12-LOX in mammalian pancreas development has not yet been identified, though loss of *Alox15* specifically in the pancreas had no effect on beta-cell mass or islet function in mice [27]. Treatment of GPR31-transfected CHO cells with its ligand 12-HETE activates the MEK and ERK pathways [46], which are involved in the regulation of cell proliferation. GPR31 is expressed in mouse and human islets and in the mouse immortalized beta-cell line βTC3. Treatment of islets from *Gpr3^1−/−^* mice with an insulin receptor inhibitor (mimicking insulin resistance) showed increased expression of beta-cell identity genes and decreased expression of ER stress genes, implicating this pathway in the regulation of beta-cell function [47].

The AA metabolites generated by 12-LOX are almost uniformly thought to be proinflammatory [48,49,50]. Because 12-LOX is expressed in metabolically-active tissues including hepatocytes, adipose tissue, and islets, it has been investigated in the context of diabetes and obesity. 12-LOX increases with age in pancreatic islets from db/db mice, a model of T2D, as is the primary 12-LOX product 12-HETE, paralleling the development of hyperglycemia and loss of islet number that is characteristic of this mouse model [51]. 12-LOX is also upregulated in islets from human donors with T2D, though immunofluorescence for insulin and 12-LOX suggests that this upregulation may be localized to PP cells rather than beta cells [52]. However, it is possible that increased 12-LOX in PP cells could still affect beta cells, since products secreted from PP cells can have paracrine and/or distal effects. Whether upregulation of 12-LOX affects secretion from PP cells, and how that might affect beta cells is not known.

Because 12-HETE and 12-LOX are increased in proinflammatory conditions and by treatment with cytokines such as IL-1β [16,28,29], the effects of 12-LOX inhibition on beta-cell mass and function have been examined under these conditions. For example, studies of global *Alox15^−/−^* mice reveal that they are resistant to streptozotocin (STZ)-induced diabetes. Islets isolated from *Alox15^−/−^* mice also have improved GSIS in the presence of cytokines [28]. Further studies in the non-obese diabetic (NOD) mouse, a model of spontaneous autoimmune diabetes, showed that loss of 12-LOX dramatically decreased diabetes incidence in both males and females [53]. These mice had preserved beta-cell mass, and decreased islet inflammation and immune infiltration [53,54]. Given that these studies were performed in mice with a global deletion of *Alox15*, the beneficial effects on beta cells may be due in part to the loss of 12-LOX function in other cell types involved in T1D etiology such as macrophages. Loss of *Alox15* in macrophages has been shown in other studies to alter their function and decrease the production of proinflammatory 12-LOX products [54,55]. However, 12-LOX expression in islets has been shown to increase with age in NOD mice [54], suggesting a contribution of locally produced pro-inflammatory 12-LOX products in islet dysfunction and destruction independent of the immune system. Indeed, similar to what was observed in the whole body knockout, specific inactivation of *Alox15* in the pancreas using Pdx1-Cre was protective against STZ-induced diabetes and improved glucose homeostasis during high-fat diet (HFD) feeding [27]. In both the STZ and HFD models, beta-cell mass was preserved in 12-LOX pancreas-specific knockout mice. This protective effect of the loss of 12-LOX function may be mediated in part by reductions in oxidative stress within beta cells due to increased expression of genes encoding antioxidant enzymes including *Gpx1* and *Nfe2l2* (Nrf2) [27,56,57]. Pharmacological inhibition of 12-LOX activity in NOD mice and in isolated mouse and human islets recapitulated the phenotypes seen in genetic inactivation models including improved GSIS and decreased cell death after cytokine treatment [29].

Other studies have examined the direct effects of treatment with lipoxygenase products on islet function and cell survival. Treatment of human islets with 12-HETE attenuates GSIS [17,29], perhaps by impairing cellular metabolic activity [17]. 12-HETE treatment alone also increased cell death in isolated human islets [17]; a similar increase in beta-cell death and decrease in cellular metabolic activity was observed in a mouse beta-cell line following 12-HETE treatment [16]. Little is known about the pathways mediating these effects; however, 12-HETE treatment has been shown to induce expression of NADPH oxidase 1 (NOX-1) in human islets [56], which could result in a prolonged increase in ROS production, leading to beta-cell dysfunction and death. Additionally, 12-HETE can activate the p38-MAPK pathway [17,58]. This pathway is known to regulate pro-inflammatory cytokine production [59], and activation by 12-HETE in beta cells may initiate or exacerbate inflammation and impair beta-cell function. Interestingly, studies in pancreatic cancer cell lines suggest that 12-HETE can activate ERK pathways to stimulate proliferation [58], but this has not been shown to occur in beta cells, suggesting a possible cell type specificity of the effects of 12-HETE within the pancreas.

## 3. Cytochrome P450-Derived Metabolites (EETs)

Cytochrome P450 (CYP) enzymes metabolize AA to produce 16-, 17-, 18-, 19-, and 20-HETE, or epoxyeicosatrienoic acids (EETs) including 5,6-, 8,9-, 11,12-, and 14,15-EET (Figure 1). There are a large number of CYP isoforms, with CYP4 isoforms generating HETEs and CYP2 isoforms producing EETs. While the effects of the LOX and COX pathways on beta-cell mass and function have been well-studied, much less is known about the effects of the products of the CYP enzymes.

In contrast to the lipoxygenase-generated 12-HETE, which attenuates GSIS, CYP-generated 20-HETE appears to act as part of a positive feedback loop to promote GSIS. Tunaru et al. demonstrated a glucose-dependent increase in 20-HETE generation and in *CYP4* isoform expression from isolated mouse and human islets, and this was blocked by a CYP inhibitor [18]. Treatment of islets with 20-HETE augmented GSIS through its activation of the fatty acid receptor FFAR1 [18]. Interestingly, this feedback loop was blunted in islets from HFD-fed mice and from human donors with T2D, implicating this pathway as a potential target for treatments to improve GSIS.

EETs have been studied widely in the context of cardiovascular and renal diseases [60,61,62,63], but less is known about their effects on beta-cell function and mass. Falck et al. demonstrated that treatment of isolated rat islets with 5,6-EET stimulated insulin release in a concentration dependent manner, while treatment with 8,9-, 11,12-, or 14,15-EET stimulated glucagon production [19]. *Cyp2j* expression has been detected specifically within whole human and rat pancreatic islets [64]; searches of publicly available data show a low level of *Cyp2j* isoform expression within alpha, beta, and delta cells [65]. Endogenous islet EET production was also detected in human and rat islets, though not all potential EET products could be quantified because of rapid hydrolysis [64]. Treatment of a rat beta-cell line with a mixture of exogenous EETs (8,9-, 11,12-, and 14,15-EET) attenuated cytokine-mediated cell death, implicating EETs in promoting beta-cell survival [20]. This treatment also resulted in a reduction in NFκB activity, which may partly explain the mechanism by which EETs promote beta-cell survival. These data support an anti-inflammatory role for EETs, consistent with their effects in other tissues [60,66].

It is difficult to study the effects of EETs particularly in vivo because of their rapid hydrolyzation by soluble epoxide hydrolase (sEH) to dihydroxyeicosatrienoic acids (DHETs), which are less biologically active. Therefore, an alternate method to study the biological functions of EETs has been to inhibit sEH activity. Loss of sEH, either through gene inactivation or through pharmacological inhibition, significantly blunted hyperglycemia in mice treated with STZ and increased GSIS in isolated islets and in vivo [30]. This improvement in GSIS may be due in part to an elevation in the intracellular calcium concentrations seen with loss of sEH activity in beta cells following glucose administration [30]. Loss of sEH was also protective against beta-cell apoptosis in mice treated with STZ, further implicating EETs as playing a role in beta-cell survival [30].

Less is known about the effects of modulating EET-producing CYP isoform expression or activity on beta cells. Loss of *Cyp2c44* (the gene encoding Cyp2c) in mice resulted in a large increase in GSIS in isolated islets, but this response was significantly blunted when GSIS was examined in vivo during a hyperglycemic clamp [31]. Further study will be needed to resolve the conflict between these in vitro and in vivo data, and to determine the effects of the loss of Cyp2c on EET levels and beta-cell function.

One area of interest is the possible role for beta-cell CYP1a isoforms in response to environmental pollutants, as chronic exposure to persistent organic pollutants (POPs) is associated with increased risk of diabetes [67,68,69], and treatment of isolated islets with the POP dioxin causes reduced insulin secretion [70,71]. Dioxin induces expression of *CYP1A1* in human islets ex vivo, and in mouse islets after in vivo systemic treatment [32]. Dioxin treatment suppresses GSIS in islets from wild type mice, but not in islets from *Cyp1a1/1a2* knockout mice, suggesting that CYP1a1 activity is involved in the effects of dioxin on insulin secretion [32]. Basal insulin secretion was also decreased in *Cyp1a1/1a2* null islets, suggesting that these enzymes act to regulate basal insulin secretion as well as GSIS [32]. Dioxin treatment also leads to an increase in beta-cell death [32]. The effects of this treatment are long-lasting, as female mice treated with dioxin showed impairments in glucose metabolism as long as 28 weeks after exposure [72]. Effects of dioxin appear to be partially sex-specific; male mice display a decrease in beta-cell mass post-dioxin exposure while female mice do not [73]. Intriguingly, HFD feeding had a similar effect of increasing *Cyp1a1* expression in islets as did dioxin exposure, implicating CYP1a1 in the response to HFD as well as pollutant exposure [74]. The precise role of CYP1a1 and its metabolites in modulating beta-cell function and mass remains to be elucidated.

## 4. Cyclooxygenase-Derived Metabolites (Prostanoids)

Cyclooxygenase (COX) 1 and 2, also known as prostaglandin-endoperoxide synthase (PTGS) 1 and 2, catalyze the conversion of AA to prostaglandin G_2_, which is rapidly converted to prostaglandin H_2_ (PGH_2_). PGH_2_ can then be converted into several bioactive molecules by a number of prostanoid synthase enzymes, generally named for their products. The principal metabolites of PGH_2_ are prostaglandin D_2_ (produced by prostaglandin D synthase (PGDS)), prostaglandin E_2_ (produced by prostaglandin E synthase (PGES)), prostaglandin F_2_ (produced by prostaglandin F synthase (PGFS)), prostaglandin I_2_ (also known as prostacyclin, produced by prostacyclin synthase), and thromboxane A_2_ (produced by thromboxane synthase (TxAS)) (Figure 1).

COX1 is thought to be constitutively expressed and active, while COX2 expression is generally thought to be induced in response to stimuli such as inflammation. However, the beta cell is somewhat unique in possessing constitutive COX2 activity that can be further upregulated by stimuli such as pro-inflammatory cytokines, high glucose levels, high free fatty acids, and PGE_2_ itself. Elevated COX2 (and COX1) expression has been documented in islets isolated from mouse models of obesity and diabetes as well as from cadaveric human organ donors with diabetes [22,25,75], which has been correlated with enhanced PGE_2_ release and/or increased plasma PGE metabolite levels [23,76]. COX2 expression in isolated islets is also positively correlated with body mass index (BMI) in non-diabetic human donors [76]. Additionally, palmitate treatment of isolated human islets increased expression of COX2, but decreasing COX2 expression in MIN6 immortalized beta cells by siRNA knockdown or by treatment with an antagonist protects against palmitate-induced apoptosis [75]. These findings suggest that COX2 and its metabolites play an important role in regulating beta-cell function and death.

**Figure 2:**
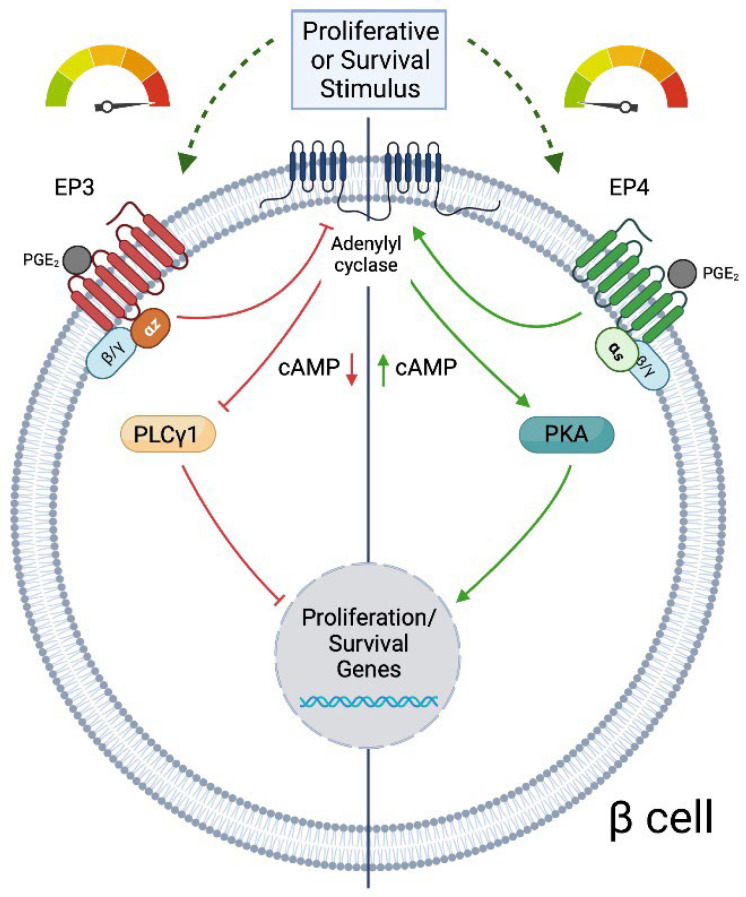
Model of EP3 and EP4 signaling in beta cells. In the presence of a proliferative or survival stimulus, when PGE_2_ is bound, EP3 and EP4 exert opposing effects on adenylyl cyclase, with EP3 inhibiting cyclic AMP (cAMP) production through Gα_z_ signaling and EP4 promoting cAMP production through G_s_ signaling. EP3 acts through the downstream effector PLCγ1 to inhibit beta-cell proliferation and survival, while EP4 acts through PKA to promote beta-cell survival.

The principal AA metabolites generated downstream of COX activity (PGD_2_, PGE_2_, PGF_2_, PGI_2_, and TXA_2_) exert their effects through G protein coupled receptors. The G protein to which each receptor couples determines the downstream effects of these metabolites. PGD_2_ acts through its receptors DP1 or DP2, which are G_S_ and G_i/o_ coupled, respectively. PGE_2_ can signal through EP1, EP2, EP3, or EP4. EP1 is principally G_q_ coupled, EP2 and EP4 are G_s_ coupled, and EP3 is coupled to the G_i_ subfamily (Figure 2). The receptors for PGF_2_ and TXA_2_ (FP and TP, respectively) couple to G_q_, and IP, the receptor for PGI_2_, is G_s_ coupled. All of these receptors are expressed in human and rodent islets and beta cells [22,25,77,78], though there is some conflicting data for the expression of *Ptger2* (EP2) [33,77]. In INS-1 (832/3) cells, mRNA for *Ptger1* (EP1), *Ptger3* (EP3), and *Ptger4* (EP4) is present at significant levels, while in isolated islets, all four EP isoforms can be detected [22,24,33].

Of the prostanoids, PGD_2_ and PGE_2_ have been the most widely studied for their roles in the modulation of beta-cell function and mass, and thus we focus on these for the remainder of this review. Early findings revealed PGD_2_ as a potent stimulator of glucagon secretion from perfused rat pancreas, with little effect on insulin secretion [79]. However, the story of PGD_2_ is not fully clear, since overexpression of prostaglandin D synthase (L-PTDS), which would be predicted to increase PGD_2_ production, reduced glucagon secretion from the alpha-cell-derived αTC-1 line, an effect mediated exclusively by DP1 [80]. Human pancreatic expression of DP2 (also known as GPR44), on the other hand, is limited to and enriched in beta cells, so much so that radiolabeled DP2 ligands have been proposed as a novel PET tracer for quantifying beta-cell mass in vivo [21,81,82,83]. High glucose and IL-1β treatment increase PGD_2_ secretion from stellate cells in human islets, and PGD_2_ suppressed glucose-stimulated and incretin-potentiated insulin secretion by signaling through DP2 [21]. However, the DP2-specific antagonist, ADZ1981, had no effect on GSIS or incretin-dependent insulin secretion in human subjects with T2D [21], and neither PGD_2_ nor DP2-selective ligands had any effect on INS-1 (832/3) cell GSIS [24]. However, Abadpour et al. recently reported that treatment with a selective DP2 antagonist, AZ8154, improved insulin secretion from human islets treated ex vivo with high glucose and IL-1β, and reduced apoptosis in those same islets [84]. Additionally, in diabetic mice transplanted with human islets, DP2 antagonist treatment led to preserved islet graft area and function, and the treated mice showed improvements in glucose tolerance and decreased fasting glucose levels [84]. Further study will be needed to reconcile these conflicting data as well as elucidate whether PGD_2_ and its receptors play a role in the maintenance of beta-cell mass.

### Prostaglandin E_2_ Signaling

In contrast to PGD_2_, much evidence has emerged for PGE_2_ as a direct regulator of beta-cell function and mass, though the effect varies depending on the EP receptor activated. PGE_2_ binds with the strongest and equal affinity to EP3 and EP4, and with lesser affinity to EP1 and EP2 [85]. There is minimal evidence for a role for EP1 or EP2 in regulating beta-cell mass and/or function. EP1 antagonist treatment of isolated rat islets did not affect GSIS, nor did it oppose the deleterious effects of IL-1β on GSIS [34], and global loss of *Ptger1* had no effect on hyperglycemia in mice treated with STZ [33]. Similarly, global loss of *Ptger2* alone had no effect on STZ-induced hyperglycemia, but wild-type mice treated with a combination of an EP2 agonist and EP4 agonist were protected against STZ-induced hyperglycemia compared to the controls [33]. However, none of these studies directly assessed the effects of EP1 and EP2 signaling on beta-cell function or survival, and therefore these effects could be due to alterations in EP1 or EP2 signaling in other tissues.

Expression of *Ptger3* (the gene encoding EP3) in the islet is dynamically regulated in pathophysiological conditions such as prediabetes, T1D, and/or T2D. For example, *Ptger3* expression is upregulated in islets from mice or humans with T2D [10,22,23,24]. In mouse islets, PGE_2_ reduces GSIS exclusively via EP3-dependent mechanisms [22,23,24]). However, the relevance of enhanced EP3 expression to human beta-cell function and mass appears limited to T2D, where *Ptger3* expression is inversely correlated with BMI [25]. In obese, non-diabetic individuals, there is no correlation of donor BMI with *Ptger3* expression [25,76]. The improvement in beta-cell function in T2D mice associated with increased EPA consumption is correlated to a decrease in *Ptger3* gene expression, with no change in expression of other EP receptors [10].

In the beta cell, EP3 is specifically coupled to the unique inhibitory G protein alpha subunit, Gα_z_ [35,36,86,87,88] (Figure 2), and activates downstream signaling through PLC γ1 [25]. C57BL/6N or J mice lacking the Gα_z_ protein globally or specifically in beta cells, respectively, are protected from HFD-induced T2D and multiple low-dose STZ induction of diabetes [35,36]. Gα_z_-null NOD mice are similarly protected from hyperglycemia, and demonstrate increased beta-cell replication and survival compared to NOD mice with intact Gα_z_ [35]. These studies suggest that therapeutic blockade of EP3 signaling would have beneficial effects in the setting of both T1D and T2D. Furthermore, combined EP3 blockade with activation of G_s_-coupled pathways in the beta cell (for example, GLP-1 signaling) might provide additional benefit. In support of this idea, co-treatment of STZ-treated Gα_z_-null mice with a sub-therapeutic dose of the GLP-1 receptor agonist, Exendin-4, potentiates GSIS, beta-cell replication, and beta-cell survival [35,89].

EP3 plays a role in the regulation of beta-cell mass and identity in addition to beta-cell function. Systemic administration of an EP3 antagonist results in increased beta-cell mass and proliferation in db/db mice [90]. In addition, EP3 blockade reversed many of the gene expression changes that occur in db/db islets including restoration of GLP-1 receptor expression and beta-cell genes such as *insulin*, *Slc2a2* (Glut2), *Nkx6.1*, *MafA*, and *Pdx1* [90].

The EP3 receptor is unique among the PGE_2_ receptors in that it has multiple splice variants (three in mice, ~10 in humans) that differ in their ligand-dependent versus constitutive activity [91,92]. Expression of the highly constitutively-active EP3γ splice variant increases with age in islets from mice, correlating with decreased beta-cell proliferative capacity [25]. This suggests that increases in EP3 receptor activity contribute to impairments in beta-cell compensation. While no direct evidence yet exists, expression of EP3γ also appears critically important for negatively regulating GSIS, potentially as a mechanism to reduce beta-cell stress and promote increased beta-cell replication [36,88,93]. As it is impossible to competitively block the activity of a ligand-independent GPCR, confirmation of this hypothesis awaits the generation of EP3γ-specific knockout mice or an inverse agonist that can block signaling through EP3γ.

The role of EP4 in the regulation of beta-cell mass and function has not been clearly defined. While treatment with an EP4 agonist alone has no effect on beta-cell proliferation in isolated mouse or human islets, beta-cell proliferation can be stimulated in isolated human islets when combined with a blockade of EP3 signaling [25]. Additionally, treatment with an EP4 agonist protects against pro-inflammatory cytokine-mediated beta-cell death in both mouse and human islets [25]. These data indicate that targeting EP4 activation may be helpful in preserving beta-cell mass in the context of T1D, where cytokine levels and PGE_2_ levels are increased. However, there are conflicting data on this point. EP4 agonist treatment suppressed the pro-inflammatory effects of macrophages on MIN6 cells [94], and treatment with agonists for EP2 and EP4 partly protects against STZ toxicity and restores beta-cell function in mice [33], suggesting that EP4 helps protect beta-cell mass and function. On the other hand, in prediabetic NOD mice, inhibition of EP4 with an antagonist decreased IL-1 levels and reduced leukocyte infiltration into the islet [95], indicating that EP4 blockade might be protective in this context. Another study investigating EP4 in the context of T1D demonstrated that PGE_2_ signaling through EP4 induced the production of some cytokines, but also decreased the expression of TNFα [96], highlighting the complexity of the effects of PGE_2_-EP4 signaling.

EP4 has also been shown to indirectly promote insulin secretion. *Ptger2* null mice treated with an EP4 agonist showed improvements in glycemia, and were protected from STZ-induced hyperglycemia [33]. In the db/db mouse model, EP4 agonism improved glucose tolerance and insulin levels [94]. However, neither of these studies examined the direct effects of EP4 on beta-cell function. In a study of the EP receptors in INS-1 (832/3) cells, EP4-selective agonists had no effect on insulin secretion [24]. Because EP3 and EP4 bind PGE_2_ with equal affinity [85] and have opposing downstream signaling pathways, it is possible that any positive effects of signaling through EP4 may be countered by signaling through EP3, and therefore a role for EP4 may only be uncovered in specific contexts (Figure 2).

EP4 couples to G_s_, leading to increased cAMP (Figure 2) and activated PKA signaling [25]. Inactivation of Gα_s_ in beta cells of adult mice results in reduced beta-cell mass, decreased insulin secretion, and glucose intolerance [37]. These deficits are apparent as early as postnatal day 28, and are associated with decreases in beta-cell proliferation and expression of beta-cell identity genes, suggesting that signaling through Gα_s_ is required to promote beta-cell maturity and establish functional postnatal beta-cell mass [37]. Signaling through Gα_s_ may be impaired in the context of T2D; Oduori et al. recently demonstrated that persistent membrane depolarization of beta cells causes a switch from G_s_ to G_q_ signaling [97]. This would suggest that EP4 agonism would not be effective as a therapeutic to improve beta-cell function in the context of T2D, since the downstream signaling pathway is impaired, but this would need to be explored further.

## 5. Conclusions

Arachidonic acid and its metabolites have distinct and diverse effects on beta-cell mass and function. Some such as 12-HETE and 5-HETE act predominantly to inhibit beta-cell function and decrease beta-cell mass, while others such as PGE_2_ can exert positive or negative effects depending on the downstream receptor through which it signals (Table 1 and Table 2). Alteration of these metabolites and/or their downstream signaling pathways has shown promise in the treatment of both T1D and T2D, both in the regulation of beta-cell function and mass as well as immune system function. However, broad inhibition of enzymes such as COX2 or PLA_2_ would lead to loss of positive-acting metabolites as well as negative-acting ones, and thus specific targeting is needed to ensure the desired beneficial effect is achieved. Additionally, a single AA metabolite may act through opposing pathways, as with PGE_2_ signaling through EP3 and EP4. Therefore, synergistic activation of a positive-acting pathway and inhibition of a negative-acting pathway using receptor-specific compounds may be needed to achieve the maximal effects on beta-cell mass and function, as seen in studies with EP3 inhibition and GLP-1R activation. Further studies are needed to identify receptors through which many AA metabolites act as well as the signaling pathways through which they exert their effects.

## Figures and Tables

**Table 1:** Summary of known effects of AA metabolite treatment on beta cells.

Metabolite	Receptor	Effect on Beta Cells	References
12-HETE	GPR31	↓ function ↑ death	[16,17]
20-HETE	FFAR1	↑ function	[18]
5,6-EET	Unknown	↑ function	[19]
8,9-EET 11,12-EET 14,15-EET	Unknown	↓ death	[20]
PGD_2_	DP2	↑ function	[21]
PGE_2_	EP3	↓ function	[22,23,24]
	EP4	↓ death	[25]

**Table 2:** Summary of known effects of loss of genes that encode AA metabolizing enzymes or signaling pathways on beta cells.

Gene Knockout	Effect on Beta Cells	References
Alox5	↓ function ↑ mass	[26]
Alox15	↑ function ↓ death	[27,28,29]
sEH	↑ function ↓ death	[30]
Cyp2c44	↑ function	[31]
Cyp1a1/2	↑ function	[32]
Ptger1	None	[33,34]
Ptger2	None	[33]
Ga_z_	↑ function ↓ death	[35,36]
Ga_s_	↓ function ↓ mass	[37]

## References

[1] CDC National Diabetes Statistics Report. https://www.cdc.gov/diabetes/data/statistics-report/index.html.

[2] Konrad R.J., Jolly Y.C., Major C., Wolf B.A. (1992). Inhibition of phospholipase A_2_ and insulin secretion in pancreatic islets. Biochim. Biophys. Acta.

[3] Ramanadham S., Gross R.W., Han X., Turk J. (1993). Inhibition of arachidonate release by secretagogue-stimulated pancreatic islets suppresses both insulin secretion and the rise in beta-cell cytosolic calcium ion concentration. Biochemistry.

[4] Persaud S.J., Muller D., Belin V.D., Kitsou-Mylona I., Asare-Anane H., Papadimitriou A., Burns C.J., Huang G.C., Amiel S.A., Jones P.M. (2007). The role of arachidonic acid and its metabolites in insulin secretion from human islets of langerhans. Diabetes.

[5] Larsson-Nyren G., Grapengiesser E., Hellman B. (2007). Phospholipase A2 is important for glucose induction of rhythmic Ca2+ signals in pancreatic β cells. Pancreas.

[6] Metz S.A. (1991). The pancreatic islet as Rubik’s Cube. Is phospholipid hydrolysis a piece of the puzzle?. Diabetes.

[7] Gross R.W., Ramanadham S., Kruszka K.K., Han X., Turk J. (1993). Rat and human pancreatic islet cells contain a calcium ion independent phospholipase A2 activity selective for hydrolysis of arachidonate which is stimulated by adenosine triphosphate and is specifically localized to islet beta-cells. Biochemistry.

[8] Dixon G., Nolan J., McClenaghan N.H., Flatt P.R., Newsholme P. (2004). Arachidonic acid, palmitic acid and glucose are important for the modulation of clonal pancreatic beta-cell insulin secretion, growth and functional integrity. Clin. Sci..

[9] Keane D.C., Takahashi H.K., Dhayal S., Morgan N.G., Curi R., Newsholme P. (2011). Arachidonic acid actions on functional integrity and attenuation of the negative effects of palmitic acid in a clonal pancreatic β-cell line. Clin. Sci..

[10] Neuman J.C., Schaid M.D., Brill A.L., Fenske R.J., Kibbe C.R., Fontaine D.A., Sdao S.M., Brar H.K., Connors K.M., Wienkes H.N. (2017). Enriching Islet Phospholipids with Eicosapentaenoic Acid Reduces Prostaglandin E_2_ Signaling and Enhances Diabetic β-Cell Function. Diabetes.

[11] Khanapure S.P., Garvey D.S., Janero D.R., Letts L.G. (2007). Eicosanoids in inflammation: Biosynthesis, pharmacology, and therapeutic frontiers. Curr. Top. Med. Chem..

[12] Dennis E.A., Norris P.C. (2015). Eicosanoid storm in infection and inflammation. Nat. Rev. Immunol..

[13] Lone A.M., Tasken K. (2013). Proinflammatory and immunoregulatory roles of eicosanoids in T cells. Front. Immunol..

[14] Imig J.D. (2020). Eicosanoid blood vessel regulation in physiological and pathological states. Clin. Sci..

[15] Jang Y., Kim M., Hwang S.W. (2020). Molecular mechanisms underlying the actions of arachidonic acid-derived prostaglandins on peripheral nociception. J. Neuroinflammation.

[16] Chen M., Yang Z.D., Smith K.M., Carter J.D., Nadler J.L. (2005). Activation of 12-lipoxygenase in proinflammatory cytokine-mediated beta cell toxicity. Diabetologia.

[17] Ma K., Nunemaker C.S., Wu R., Chakrabarti S.K., Taylor-Fishwick D.A., Nadler J.L. (2010). 12-Lipoxygenase Products Reduce Insulin Secretion and β-Cell Viability in Human Islets. J. Clin. Endocrinol. Metab..

[18] Tunaru S., Bonnavion R., Brandenburger I., Preussner J., Thomas D., Scholich K., Offermanns S. (2018). 20-HETE promotes glucose-stimulated insulin secretion in an autocrine manner through FFAR1. Nat. Commun..

[19] Falck J.R., Manna S., Moltz J., Chacos N., Capdevila J. (1983). Epoxyeicosatrienoic acids stimulate glucagon and insulin release from isolated rat pancreatic islets. Biochem. Biophys. Res. Commun..

[20] Grimes D., Watson D. (2019). Epoxyeicosatrienoic acids protect pancreatic beta cells against pro-inflammatory cytokine toxicity. Biochem. Biophys. Res. Commun..

[21] Skrtic S., Tyrberg B., Broberg M., Ericsson H., Schnecke V., Kjaer M., Hompesch M., Andersson E.M., Ryberg E., Aivazidis A. (2018). Exploring the insulin secretory properties of the PGD_2_-GPR44/DP2 axis in vitro and in a randomized phase-1 trial of type 2 diabetes patients. PLoS ONE.

[22] Kimple M.E., Keller M.P., Rabaglia M.R., Pasker R.L., Neuman J.C., Truchan N.A., Brar H.K., Attie A.D. (2013). Prostaglandin E_2_ receptor, EP3, is induced in diabetic islets and negatively regulates glucose- and hormone-stimulated insulin secretion. Diabetes.

[23] Schaid M.D., Zhu Y., Richardson N.E., Patibandla C., Ong I.M., Fenske R.J., Neuman J.C., Guthery E., Reuter A., Sandhu H.K. (2021). Systemic Metabolic Alterations Correlate with Islet-Level Prostaglandin E_2_ Production and Signaling Mechanisms That Predict β-Cell Dysfunction in a Mouse Model of Type 2 Diabetes. Metabolites.

[24] Sandhu H.K., Neuman J.C., Schaid M.D., Davis S.E., Connors K.M., Challa R., Guthery E., Fenske R.J., Patibandla C., Breyer R.M. (2021). Rat prostaglandin EP3 receptor is highly promiscuous and is the sole prostanoid receptor family member that regulates INS-1 (832/3) cell glucose-stimulated insulin secretion. Pharmacol. Res. Perspect..

[25] Carboneau B.A., Allan J.A., Townsend S.E., Kimple M.E., Breyer R.M., Gannon M. (2017). Opposing effects of prostaglandin E_2_ receptors EP3 and EP4 on mouse and human β-cell survival and proliferation. Mol. Metab..

[26] Mehrabian M., Schulthess F.T., Nebohacova M., Castellani L.W., Zhou Z., Hartiala J., Oberholzer J., Lusis A.J., Maedler K., Allayee H. (2008). Identification of ALOX_5_ as a gene regulating adiposity and pancreatic function. Diabetologia.

[27] Tersey S.A., Maier B., Nishiki Y., Maganti A.V., Nadler J.L., Mirmira R.G. (2014). 12-lipoxygenase promotes obesity-induced oxidative stress in pancreatic islets. Mol. Cell. Biol..

[28] Bleich D., Chen S., Zipser B., Sun D., Funk C.D., Nadler J.L. (1999). Resistance to type 1 diabetes induction in 12-lipoxygenase knockout mice. J. Clin. Investig..

[29] Taylor-Fishwick D.A., Weaver J., Glenn L., Kuhn N., Rai G., Jadhav A., Simeonov A., Dudda A., Schmoll D., Holman T.R. (2015). Selective inhibition of 12-lipoxygenase protects islets and beta cells from inflammatory cytokine-mediated beta cell dysfunction. Diabetologia.

[30] Luo P., Chang H.H., Zhou Y., Zhang S., Hwang S.H., Morisseau C., Wang C.Y., Inscho E.W., Hammock B.D., Wang M.H. (2010). Inhibition or deletion of soluble epoxide hydrolase prevents hyperglycemia, promotes insulin secretion, and reduces islet apoptosis. J. Pharmacol. Exp. Ther..

[31] Gangadhariah M.H., Dieckmann B.W., Lantier L., Kang L., Wasserman D.H., Chiusa M., Caskey C.F., Dickerson J., Luo P., Gamboa J.L. (2017). Cytochrome P450 epoxygenase-derived epoxyeicosatrienoic acids contribute to insulin sensitivity in mice and in humans. Diabetologia.

[32] Ibrahim M., MacFarlane E.M., Matteo G., Hoyeck M.P., Rick K.R.C., Farokhi S., Copley C.M., O’Dwyer S., Bruin J.E. (2020). Functional cytochrome P450 1A enzymes are induced in mouse and human islets following pollutant exposure. Diabetologia.

[33] Vennemann A., Gerstner A., Kern N., Bouzas N.F., Narumiya S., Maruyama T., Nusing R.M. (2012). PTGS-2-PTGER2/4 signaling pathway partially protects from diabetogenic toxicity of streptozotocin in mice. Diabetes.

[34] Tran P.O., Gleason C.E., Robertson R.P. (2002). Inhibition of interleukin-1β-induced COX-2 and EP3 gene expression by sodium salicylate enhances pancreatic islet beta-cell function. Diabetes.

[35] Fenske R.J., Cadena M.T., Harenda Q.E., Wienkes H.N., Carbajal K., Schaid M.D., Laundre E., Brill A.L., Truchan N.A., Brar H. (2017). The Inhibitory G Protein alpha-Subunit, Galphaz, Promotes Type 1 Diabetes-Like Pathophysiology in NOD Mice. Endocrinology.

[36] Schaid M.D., Green C.L., Peter D.C., Gallagher S.J., Guthery E., Carbajal K.A., Harrington J.M., Kelly G.M., Reuter A., Wehner M.L. (2021). Agonist-independent Gα_z_ activity negatively regulates beta-cell compensation in a diet-induced obesity model of type 2 diabetes. J. Biol. Chem..

[37] Serra-Navarro B., Fernandez-Ruiz R., Garcia-Alaman A., Pradas-Juni M., Fernandez-Rebollo E., Esteban Y., Mir-Coll J., Mathieu J., Dalle S., Hahn M. (2021). Gsα-dependent signaling is required for postnatal establishment of a functional β-cell mass. Mol. Metab..

[38] Tersey S.A., Bolanis E., Holman T.R., Maloney D.J., Nadler J.L., Mirmira R.G. (2015). Minireview: 12-Lipoxygenase and Islet β-Cell Dysfunction in Diabetes. Mol. Endocrinol..

[39] Camara N.O., Martins J.O., Landgraf R.G., Jancar S. (2009). Emerging roles for eicosanoids in renal diseases. Curr. Opin. Nephrol. Hypertens..

[40] Hao C.M., Breyer M.D. (2007). Physiologic and pathophysiologic roles of lipid mediators in the kidney. Kidney Int..

[41] Helgadottir A., Manolescu A., Thorleifsson G., Gretarsdottir S., Jonsdottir H., Thorsteinsdottir U., Samani N.J., Gudmundsson G., Grant S.F., Thorgeirsson G. (2004). The gene encoding 5-lipoxygenase activating protein confers risk of myocardial infarction and stroke. Nat. Genet..

[42] Mehrabian M., Allayee H. (2003). 5-lipoxygenase and atherosclerosis. Curr. Opin. Lipidol..

[43] Poeckel D., Funk C.D. (2010). The 5-lipoxygenase/leukotriene pathway in preclinical models of cardiovascular disease. Cardiovasc. Res..

[44] Turk J., Colca J.R., Kotagal N., McDaniel M.L. (1984). Arachidonic acid metabolism in isolated pancreatic islets. II. The effects of glucose and of inhibitors of arachidonate metabolism on insulin secretion and metabolite synthesis. Biochim. Biophys. Acta.

[45] Hernandez-Perez M., Kulkarni A., Samala N., Sorrell C., El K., Haider I., Aleem A.M., Holman T.R., Rai G., Tersey S.A. (2020). A 12-lipoxygenase-Gpr31 signaling axis is required for pancreatic organogenesis in the zebrafish. FASEB J..

[46] Guo Y., Zhang W., Giroux C., Cai Y., Ekambaram P., Dilly A.K., Hsu A., Zhou S., Maddipati K.R., Liu J. (2011). Identification of the orphan G protein-coupled receptor GPR31 as a receptor for 12-(S)-hydroxyeicosatetraenoic acid. J. Biol. Chem..

[47] Hernandez-Perez M., Haider I., Anderson R.M., Tersey S.A., Mirmira R. (2020). 2111-P: Role of G-Protein Coupled Receptor 31 (GPR31) in ß-Cell Health and Disease. Diabetes.

[48] Kuhn H., O’Donnell V.B. (2006). Inflammation and immune regulation by 12/15-lipoxygenases. Prog. Lipid Res..

[49] Middleton M.K., Rubinstein T., Pure E. (2006). Cellular and molecular mechanisms of the selective regulation of IL-12 production by 12/15-lipoxygenase. J. Immunol..

[50] Kulkarni A., Nadler J.L., Mirmira R.G., Casimiro I. (2021). Regulation of Tissue Inflammation by 12-Lipoxygenases. Biomolecules.

[51] Dobrian A.D., Huyck R.W., Glenn L., Gottipati V., Haynes B.A., Hansson G.I., Marley A., McPheat W.L., Nadler J.L. (2018). Activation of the 12/15 lipoxygenase pathway accompanies metabolic decline in db/db pre-diabetic mice. Prostaglandins Other Lipid Mediat..

[52] Grzesik W.J., Nadler J.L., Machida Y., Nadler J.L., Imai Y., Morris M.A. (2015). Expression pattern of 12-lipoxygenase in human islets with type 1 diabetes and type 2 diabetes. J. Clin. Endocrinol. Metab..

[53] McDuffie M., Maybee N.A., Keller S.R., Stevens B.K., Garmey J.C., Morris M.A., Kropf E., Rival C., Ma K., Carter J.D. (2008). Nonobese diabetic (NOD) mice congenic for a targeted deletion of 12/15-lipoxygenase are protected from autoimmune diabetes. Diabetes.

[54] Green-Mitchell S.M., Tersey S.A., Cole B.K., Ma K., Kuhn N.S., Cunningham T.D., Maybee N.A., Chakrabarti S.K., McDuffie M., Taylor-Fishwick D.A. (2013). Deletion of 12/15-lipoxygenase alters macrophage and islet function in NOD-*Alox15^null^* mice, leading to protection against type 1 diabetes development. PLoS ONE.

[55] Kulkarni A., Pineros A.R., Walsh M.A., Casimiro I., Ibrahim S., Hernandez-Perez M., Orr K.S., Glenn L., Nadler J.L., Morris M.A. (2021). 12-Lipoxygenase governs the innate immune pathogenesis of islet inflammation and autoimmune diabetes. JCI Insight.

[56] Weaver J.R., Holman T.R., Imai Y., Jadhav A., Kenyon V., Maloney D.J., Nadler J.L., Rai G., Simeonov A., Taylor-Fishwick D.A. (2012). Integration of pro-inflammatory cytokines, 12-lipoxygenase and NOX-1 in pancreatic islet beta cell dysfunction. Mol. Cell. Endocrinol..

[57] Conteh A.M., Reissaus C.A., Hernandez-Perez M., Nakshatri S., Anderson R.M., Mirmira R.G., Tersey S.A., Linnemann A.K. (2019). Platelet-type 12-lipoxygenase deletion provokes a compensatory 12/15-lipoxygenase increase that exacerbates oxidative stress in mouse islet β cells. J. Biol. Chem..

[58] Ding X.Z., Tong W.G., Adrian T.E. (2001). 12-lipoxygenase metabolite 12(S)-HETE stimulates human pancreatic cancer cell proliferation via protein tyrosine phosphorylation and ERK activation. Int. J. Cancer.

[59] Cuenda A., Rousseau S. (2007). p38 MAP-kinases pathway regulation, function and role in human diseases. Biochim. Biophys. Acta.

[60] Imig J.D. (2005). Epoxide hydrolase and epoxygenase metabolites as therapeutic targets for renal diseases. Am. J. Physiol. Ren. Physiol..

[61] Deng Y., Theken K.N., Lee C.R. (2010). Cytochrome P450 epoxygenases, soluble epoxide hydrolase, and the regulation of cardiovascular inflammation. J. Mol. Cell. Cardiol..

[62] Yang L., Maki-Petaja K., Cheriyan J., McEniery C., Wilkinson I.B. (2015). The role of epoxyeicosatrienoic acids in the cardiovascular system. Br. J. Clin. Pharmacol..

[63] Sonnweber T., Pizzini A., Nairz M., Weiss G., Tancevski I. (2018). Arachidonic Acid Metabolites in Cardiovascular and Metabolic Diseases. Int. J. Mol. Sci..

[64] Zeldin D.C., Foley J., Boyle J.E., Moomaw C.R., Tomer K.B., Parker C., Steenbergen C., Wu S. (1997). Predominant expression of an arachidonate epoxygenase in islets of Langerhans cells in human and rat pancreas. Endocrinology.

[65] DiGruccio M.R., Mawla A.M., Donaldson C.J., Noguchi G.M., Vaughan J., Cowing-Zitron C., van der Meulen T., Huising M.O. (2016). Comprehensive alpha, beta and delta cell transcriptomes reveal that ghrelin selectively activates delta cells and promotes somatostatin release from pancreatic islets. Mol. Metab..

[66] Thomson S.J., Askari A., Bishop-Bailey D. (2012). Anti-inflammatory effects of epoxyeicosatrienoic acids. Int. J. Vasc. Med..

[67] Calvert G.M., Sweeney M.H., Deddens J., Wall D.K. (1999). Evaluation of diabetes mellitus, serum glucose, and thyroid function among United States workers exposed to 2,3,7,8-tetrachlorodibenzo-p-dioxin. Occup. Environ. Med..

[68] Bertazzi P.A., Consonni D., Bachetti S., Rubagotti M., Baccarelli A., Zocchetti C., Pesatori A.C. (2001). Health effects of dioxin exposure: A 20-year mortality study. Am. J. Epidemiol..

[69] Wu H., Bertrand K.A., Choi A.L., Hu F.B., Laden F., Grandjean P., Sun Q. (2013). Persistent organic pollutants and type 2 diabetes: A prospective analysis in the nurses’ health study and meta-analysis. Environ. Health Perspect..

[70] Novelli M., Piaggi S., De Tata V. (2005). 2,3,7,8-Tetrachlorodibenzo-p-dioxin-induced impairment of glucose-stimulated insulin secretion in isolated rat pancreatic islets. Toxicol. Lett..

[71] Lee Y.M., Ha C.M., Kim S.A., Thoudam T., Yoon Y.R., Kim D.J., Kim H.C., Moon H.B., Park S., Lee I.K. (2017). Low-Dose Persistent Organic Pollutants Impair Insulin Secretory Function of Pancreatic β-Cells: Human and In Vitro Evidence. Diabetes.

[72] Hoyeck M.P., Merhi R.C., Blair H.L., Spencer C.D., Payant M.A., Alfonso D.I.M., Zhang M., Matteo G., Chee M.J., Bruin J.E. (2020). Female mice exposed to low doses of dioxin during pregnancy and lactation have increased susceptibility to diet-induced obesity and diabetes. Mol. Metab..

[73] Hoyeck M.P., Blair H., Ibrahim M., Solanki S., Elsawy M., Prakash A., Rick K.R.C., Matteo G., O’Dwyer S., Bruin J.E. (2020). Long-term metabolic consequences of acute dioxin exposure differ between male and female mice. Sci. Rep..

[74] Matteo G., Hoyeck M.P., Blair H.L., Zebarth J., Rick K.R.C., Williams A., Gagne R., Buick J.K., Yauk C.L., Bruin J.E. (2021). Prolonged Low-Dose Dioxin Exposure Impairs Metabolic Adaptability to High-Fat Diet Feeding in Female but Not Male Mice. Endocrinology.

[75] Amior L., Srivastava R., Nano R., Bertuzzi F., Melloul D. (2019). The role of Cox-2 and prostaglandin E_2_ receptor EP3 in pancreatic β-cell death. FASEB J..

[76] Truchan N.A., Fenske R.J., Sandhu H.K., Weeks A.M., Patibandla C., Wancewicz B., Pabich S., Reuter A., Harrington J.M., Brill A.L. (2021). Human Islet Expression Levels of Prostaglandin E_2_ Synthetic Enzymes, But Not Prostaglandin EP3 Receptor, Are Positively Correlated with Markers of β-Cell Function and Mass in Nondiabetic Obesity. ACS Pharmacol. Transl. Sci..

[77] Ku G.M., Kim H., Vaughn I.W., Hangauer M.J., Myung Oh C., German M.S., McManus M.T. (2012). Research resource: RNA-Seq reveals unique features of the pancreatic β-cell transcriptome. Mol. Endocrinol..

[78] Bramswig N.C., Everett L.J., Schug J., Dorrell C., Liu C., Luo Y., Streeter P.R., Naji A., Grompe M., Kaestner K.H. (2013). Epigenomic plasticity enables human pancreatic α to β cell reprogramming. J. Clin. Investig..

[79] Horie H., Matsuyama T., Namba M., Nonaka K., Tarui S. (1983). Modulation by prostaglandin D_2_ of glucagon and insulin secretion in the perfused rat pancreas. Prostaglandins Leukot. Med..

[80] Davani D., Kumar S., Palaia T., Hall C., Ragolia L. (2015). Lipocalin-type prostaglandin D_2_ synthase reduces glucagon secretion in alpha TC-1 clone 6 cells via the DP1 receptor. Biochem. Biophys. Rep..

[81] Hellstrom-Lindahl E., Danielsson A., Ponten F., Czernichow P., Korsgren O., Johansson L., Eriksson O. (2016). GPR44 is a pancreatic protein restricted to the human beta cell. Acta Diabetol..

[82] Jahan M., Johnstrom P., Selvaraju R.K., Svedberg M., Winzell M.S., Bernstrom J., Kingston L., Schou M., Jia Z., Skrtic S. (2018). The development of a GPR44 targeting radioligand [^11^C]AZ12204657 for in vivo assessment of beta cell mass. EJNMMI Res..

[83] Eriksson O., Johnstrom P., Cselenyi Z., Jahan M., Selvaraju R.K., Jensen-Waern M., Takano A., Winzell M.S., Halldin C., Skrtic S. (2018). In Vivo Visualization of β-Cells by Targeting of GPR44. Diabetes.

[84] Abadpour S., Tyrberg B., Schive S.W., Huldt C.W., Gennemark P., Ryberg E., Ryden-Bergsten T., Smith D.M., Korsgren O., Skrtic S. (2020). Inhibition of the prostaglandin D_2_-GPR_44_/DP_2_ axis improves human islet survival and function. Diabetologia.

[85] Abramovitz M., Adam M., Boie Y., Carriere M., Denis D., Godbout C., Lamontagne S., Rochette C., Sawyer N., Tremblay N.M. (2000). The utilization of recombinant prostanoid receptors to determine the affinities and selectivities of prostaglandins and related analogs. Biochim. Biophys. Acta.

[86] Kimple M.E., Nixon A.B., Kelly P., Bailey C.L., Young K.H., Fields T.A., Casey P.J. (2005). A role for G_z_ in pancreatic islet β-cell biology. J. Biol. Chem..

[87] Kimple M.E., Moss J.B., Brar H.K., Rosa T.C., Truchan N.A., Pasker R.L., Newgard C.B., Casey P.J. (2012). Deletion of Gα_Z_ protein protects against diet-induced glucose intolerance via expansion of β-cell mass. J. Biol. Chem..

[88] Wisinski J.A., Reuter A., Peter D.C., Schaid M.D., Fenske R.J., Kimple M.E. (2021). Prostaglandin EP3 receptor signaling is required to prevent insulin hypersecretion and metabolic dysfunction in a non-obese mouse model of insulin resistance. Am. J. Physiol. Endocrinol. Metab..

[89] Brill A.L., Wisinski J.A., Cadena M.T., Thompson M.F., Fenske R.J., Brar H.K., Schaid M.D., Pasker R.L., Kimple M.E. (2016). Synergy between Gα_z_ Deficiency and GLP-1 Analog Treatment in Preserving Functional β-Cell Mass in Experimental Diabetes. Mol. Endocrinol..

[90] Bosma K.J., Andrei S.R., Katz L.S., Smith A.A., Dunn J.C., Ricciardi V.F., Ramirez M.A., Baumel-Alterzon S., Pace W.A., Carroll D.T. (2021). Pharmacological blockade of the EP3 prostaglandin E_2_ receptor in the setting of type 2 diabetes enhances β-cell proliferation and identity and relieves oxidative damage. Mol. Metab..

[91] Hasegawa H., Negishi M., Ichikawa A. (1996). Two isoforms of the prostaglandin E receptor EP3 subtype different in agonist-independent constitutive activity. J. Biol. Chem..

[92] Tomasch M., Schwed J.S., Kuczka K., Dos Santos S.M., Harder S., Nusing R.M., Paulke A., Stark H. (2012). Fluorescent Human EP3 Receptor Antagonists. ACS Med. Chem. Lett..

[93] Kimple M.E., Joseph J.W., Bailey C.L., Fueger P.T., Hendry I.A., Newgard C.B., Casey P.J. (2008). Gα_z_ negatively regulates insulin secretion and glucose clearance. J. Biol. Chem..

[94] Yasui-Kato M., Patlada S., Yokode M., Kamei K., Minami M. (2020). EP4 signalling is essential for controlling islet inflammation by causing a shift in macrophage polarization in obesity/type 2 diabetes. Diab. Vasc. Dis. Res..

[95] Rahman M.J., Rodrigues K.B., Quiel J.A., Liu Y., Bhargava V., Zhao Y., Hotta-Iwamura C., Shih H.Y., Lau-Kilby A.W., Malloy A.M. (2018). Restoration of the type I IFN-IL-1 balance through targeted blockade of PTGER4 inhibits autoimmunity in NOD mice. JCI Insight.

[96] Vallerie S.N., Kramer F., Barnhart S., Kanter J.E., Breyer R.M., Andreasson K.I., Bornfeldt K.E. (2016). Myeloid Cell Prostaglandin E_2_ Receptor EP4 Modulates Cytokine Production but Not Atherogenesis in a Mouse Model of Type 1 Diabetes. PLoS ONE.

[97] Oduori O.S., Murao N., Shimomura K., Takahashi H., Zhang Q., Dou H., Sakai S., Minami K., Chanclon B., Guida C. (2020). Gs/Gq signaling switch in β cells defines incretin effectiveness in diabetes. J. Clin. Investig..

